# Supplementary material for: Additive rheology of complex granular flows
Source: Nat Commun. 2020 Mar 19;11:1476. doi: 10.1038/s41467-020-15263-3 (PMC7081210; doi:10.1038/s41467-020-15263-3)
Supplement: Supplementary file 1 — Supplementary Information [file 41467_2020_15263_MOESM1_ESM.pdf]

Supplementary Information
Additive rheology of complex granular flows

Vo et al.

Supplementary Note 1

The rheology of complex granular flows investigated in this paper is based on careful measurements of the flow variables and microstructural parameters in the steady flowing state. Although shear-banding (strain localization) can occur in granular materials, it is essential to avoid shear banding for the investigation of the rheological behavior by an appropriate choice of the boundary and driving conditions. We find that the wall roughness, flow thickness and inertial number determine the velocity profiles. Supplementary Figures 1 and 2 display examples of velocity profiles in cohesive and cohesionless flows. We see that in all cases the granular material is sheared in its whole volume, but the velocity profiles are not linear and the material may slip at the walls. This implies that, in general, the average shear rates $\dot{\gamma}$ can not be simply calculated from the wall displacement rate but should account also for wall slip. This is what we did for the calculation of $\dot{\gamma}$. The shear rates used in the paper are average values over the whole sample.

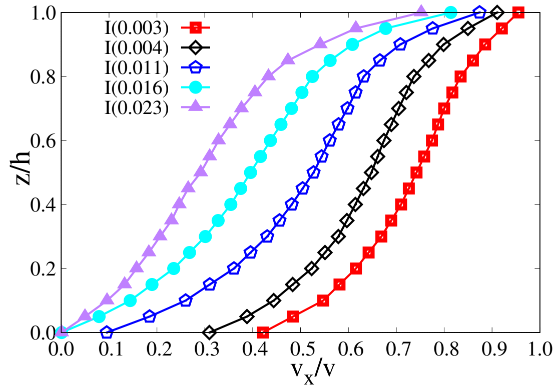

Supplementary Figure 1: **Velocity profiles for different values of the inertial number**. The cohesion index is $\xi = 0.5$ and liquid viscosity is $\eta_f = 1$ mPa.s. The velocities are average values in the steady-state flow and they are normalized by the shearing velocity of the top wall.

Supplementary Note 2

The microstructure in the quasistatic limit $I_m \to 0$ is characterized by the coordination number Z_c and bond orientation anisotropy A_c. Both Z_c and A_c decline almost linearly with increasing cohesion index ξ, as shown in Supplementary Fig. 3:

$$Z_c \simeq Z_0(1 - a'\xi) \quad (1)$$
$$A_c \simeq A_0(1 - b'\xi) \quad (2)$$

with $a' \simeq 0.005$ and $b' \simeq 0.083$. The data point for $\xi = 0$ corresponds to a dry sample with both cohesion and viscous force set to zero.

Note that for cohesive contacts, the capillary bonds define a network that includes both the contacts and the pairs of particles separated by a gap below the debonding distance. For the calculation of Z_c and A_c in the dry limit, we keep the same definition of the network with the same value of the gap. Hence, the coordination number $Z_0 \simeq 6.12$ at vanishing cohesion and inertia is defined for both contacts and pair of particles having a gap below the debonding distance. In the same way, the bond anisotropy $A_0 \simeq 0.07$ represents the anisotropy in the same limit and for the same network. The value of the coordination number for contact network is lower whereas the contact network anisotropy is higher compared to the bond network.

The fitting forms shown in Fig. 5 for the normalized coordination number Z/Z_c and normalized anisotropy A/A_c are the same as for Φ/Φ_c and μ/μ_c, respectively:

$$\frac{A}{A_c} = 1 + \frac{\Delta_A}{1 + I_A/I_m} \quad (3)$$

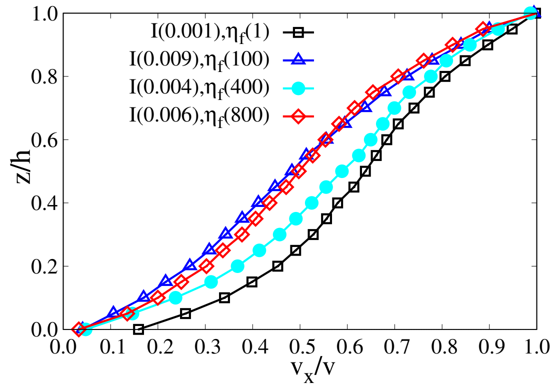

Supplementary Figure 2: **Velocity profiles for different values of the inertial number and liquid viscosity**. The unit of liquid viscosity η_f is mPa.s, and the samples are cohesionless. The velocities are average values in the steady state, and they are normalized by the shearing velocity of the top wall.

Email address: franck.radjai@umontpellier.fr (Farhang Radjai)

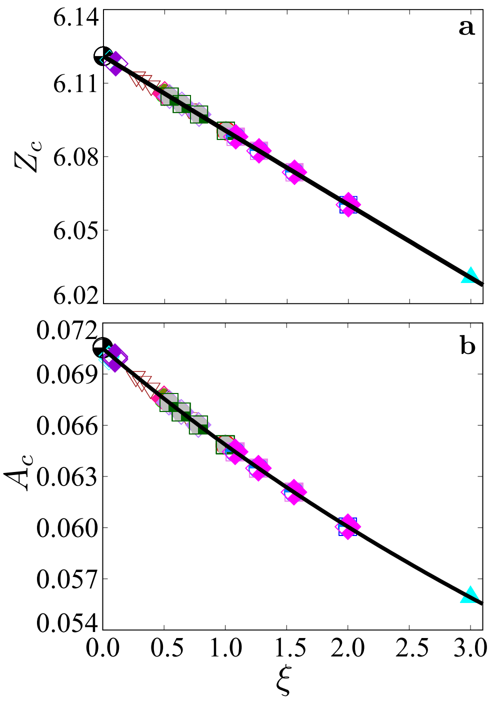

Supplementary Figure 3: **Quasistatic microstructure**. Bond coordination number Z_c (**a**) and bond orientation anisotropy A_c (**b**) in the quasi-static state as a function of the cohesion index ξ. The symbols and their colors correspond to the same sets of flow parameters as in Fig. 2.

$$\frac{Z}{Z_c} = \frac{1}{1 + I_m/I_Z} \tag{4}$$

with $\Delta_A \simeq 2.410$, $I_A \simeq 0.215$ and $I_Z \simeq 1.250$. Note that, the modified inertial number I_m is defined here with $\alpha \simeq 0.062$ and $\beta \simeq 0.075$ as for μ/μ_c and Φ/Φ_c although the fitting parameters are different.

Supplementary Note 3

We describe here in more detail the derivation of the effective viscosities as a function of the packing fraction Φ in the NVT ensemble from μ and Φ as functions of the generalized inertial number I_m in the NPT ensemble with temperature $T = 0$. This transition was presented in Supplementary Ref. [1] for dry granular flows and extended to dense inertial suspensions in Supplementary Ref. [2]. For wet granular materials, we follow the same argument by accounting for the viscous and cohesive stresses in addition to the confining and inertial stresses.

In the NVT ensemble, the packing fraction Φ replaces the normal stress σ_n as control parameter, and the rheology is characterized by the effective normal and shear viscosities, η_n and η_t, as a function of Φ. The key point is that at constant volume no external stress σ_p is imposed, and thus both the normal stress σ_n and shear stress σ_t are induced by the shear rate $\dot{\gamma}$. The effective viscosities are defined by

$$\sigma_n = \eta_n \dot{\gamma}, \tag{5}$$

$$\sigma_t = \eta_t \dot{\gamma}, \tag{6}$$

implying that σ_n and σ_t vanish when $\dot{\gamma} \to 0$.

As in the NPT ensemble, in the steady-state flow we have

$$\sigma_t = \mu \sigma_n. \tag{7}$$

Since no external stress is imposed, the shear stress σ_t should scale with the total shear-dependent internal stress $\sigma_i + \beta\sigma_v$, where β is the same factor as in the NPT ensemble. Hence, we set

$$\sigma_t = c_t(\sigma_i + \beta\sigma_v), \tag{8}$$

where c_t is a dimensionless factor. From equations (7) and (8), we get

$$\sigma_n = c_n(\sigma_i + \beta\sigma_v), \tag{9}$$

with

$$c_t = \mu c_n \tag{10}$$

Another key point is that the NPT and NVT viewpoints describe the same stress state if the normal stress σ_n in the NVT ensemble is equal to the normal stress imposed in the NPT ensemble. This stress is σ_p enhanced by the presence of the cohesive stress:

$$\sigma_n = \sigma_p + \alpha\sigma_c, \tag{11}$$

where α is the same factor as in the NPT ensemble. Equations (5), (6), (9) and (11) together with equation (1) in the paper and the expressions of the inertial number I and cohesion index ξ, lead to

$$c_n = \frac{1}{I_m^2} = \frac{\eta_n}{\beta\eta + \rho_s \langle d \rangle^2 \dot{\gamma}}, \tag{12}$$

and

$$c_t = \frac{\mu}{I_m^2} = \frac{\eta_t}{\beta\eta + \rho_s \langle d \rangle^2 \dot{\gamma}}. \tag{13}$$

These expressions indicate that c_n and c_t represent dimensionless viscosities with $\beta\eta + \rho_s\langle d \rangle^2 \dot{\gamma}$ playing the role of a reference viscosity. The first term is the liquid viscosity and the second term is the shear-induced inertial viscosity. By eliminating I_m between the analytical expressions of $\mu(I_m)$ and $\Phi(I_m)$ and from the above expressions of c_n and c_t, one arrives at the expressions of $c_n(\Phi)$ and $c_t(\Phi)$ given by equations (8) and (9).

Supplementary References

[1] F. Boyer, E. Guazzelli, O. Pouliquen, Unifying suspension and granular rheology, Phys. Rev. Lett. 107, 188301, (2011).
[2] L. Amarsid, J.-Y. Delenne, P. Mutabaruka, Y. Monerie, F. Perales, F. Radjai, Viscoinertial regime of immersed granular flows, Phys. Rev. E 96, 012901, (2017).
